# Factors Associated with Atopy in Toddlers: A Case-Control Study

**DOI:** 10.3390/ijerph120302501

**Published:** 2015-02-25

**Authors:** Jolene Yung, John W. M. Yuen, Yvonne Ou, Alice Yuen Loke

**Affiliations:** 1 School of Nursing, the Hong Kong Polytechnic University, Hong Kong, China; E-Mails: j.yung@connect.polyu.hk (J.Y.); john.yuen@polyu.edu.hk (J.W.M.Y); 2 Central Health Medical Practice, Hong Kong, China; E-Mail: drou@centralhealth.com.hk

**Keywords:** breast-feeding, maternal antibiotic use, atopy, toddlers

## Abstract

In this case-control study the association between the approaches used to feed infants, together with known family and environmental factors, and the occurrence of atopic illness in toddlers between the ages of 4 months to 3 years in Hong Kong was examined. A total of 206 subjects were recruited from April to June of 2014. The results obtained by binary logistic regression indicated that atopy is associated with boys (OR 2.072, CI 1.089–3.941), the maternal use of antibiotics *in utero* or while breast feeding (OR 2.276, CI 1.151–4.504), the later commencement of mixed feeding (OR 2.497, CI 1.025–6.082), breast feeding exclusively for 3 months (OR 1.972, CI 1.009–3.857), and having a mother who was diagnosed with eczema (OR 4.510, CI 1.764–11.530). Although an exclusive reliance on breast feeding has been shown to be predictive of atopy among toddlers, the positive qualities of breast milk cannot be ignored. A further study of the contents and nutritional values of breast milk is warranted.

## 1. Introduction

Atopic disorders, predominantly eczema and atopic dermatitis, affected up to 15 percent of Hong Kong children aged 14 and under in 2011 [1]. This is comparable to the rates reported in most Western countries, ranging from 12.5 to 23.4 percent [2,3,4], and much lower than the 25 to 40 percent reported in Australia [5].

Atopic manifestations are not apparent at birth, but usually appear in early infancy, before the age of three months. That the etiology of atopic manifestations involves both heredity and the environment has been well documented. An individual with one parent and a sibling with a history of atopic illness has a 40% chance of developing an atopic illness [5]. The environmental determinant includes everything surrounding a child, including the child’s birthplace, home, playmates, and diet. A number of benign items from the environment such as dust, pets, pollen, and so forth, have been recognized as allergens that, with contact, could lead to the manifestation of atopic illness in children. Studies have identified exposure to carpeting [6], preschool [7], dust mites [5], pets [8,9], antibiotic use in the prenatal period or in childhood [9,10], siblings [10,11], and the first exposure to solid food [12,13] as factors associated with atopic manifestations. Besides heredity and the environment, there is growing evidence that some approaches to feeding in early life are associated with the development of atopic illness.

It is well accepted that breast milk provides optimal nourishment for neonates and protection against infection and allergies [14]. Breast milk offers passive immunity to infants through secretory immunoglobulin A (IgA) antibodies that neutralize countless pathogenic microorganisms [15,16]. Breast milk also contains probiotic substances that trigger the growth of a number of beneficial microflora in the gut of infants [17]. It has been reported that breastfeeding (BF) for 3 weeks already enhances the effects of gut maturation in infants and provides protective factors against allergenic antigens [7,16].

However, within the context of atopy, some researchers now consider long durations of exclusive breastfeeding (EBF) to be a contraindication rather than a preventative measure. Some studies have reported an increased risk of eczema or atopic dermatitis [18,19,20] and asthma [21] from prolonged EBF among children aged five and under. Specifically, the risk of atopic dermatitis increased with each added month of BF [18], in circumstances of EBF for a duration of over 2 months [20] or for a longer period of 6 months [19].

A number of studies have claimed that there is no evidence to show that EBF has either a protective or harmful effect on atopic development in children [19,21,22,23]. There is also an ongoing debate about the effect of the timing of the first exposure to solid food for toddlers in relation to the development of atopy. Parents are recommended to delay exposing their toddlers to solid or complementary foods until they reach the age of 6 months [24]. However, there is recent evidence to suggest that the early introduction of solid foods is beneficial [12,13]. In 2010, in a Finnish birth cohort study [12], an increased incidence of atopy involving an allergic sensitization to food and inhalant allergens was observed of toddlers at age of five, and associated with the introduction of complementary foods at 4 months or later. The results of this study challenge the commonly recommended practice. In 2013, further evidence from the same study indicated that the risk of sensitization to various allergens was significantly reduced by the early introduction of complementary foods and the short duration of breastfeeding [13]. Similar observations were reported on the development of eczema [25,26].

Despite the debate, overall BF rates are on the decline globally. The rate at which BF is initiated is relatively high in most countries; however, there a significant decline in BF is seen the early months of infancy. In the United States, 74% of the mothers of all infants born in 2006 initiated BF, but EBF rates dropped to 34% and 14% by the ages of 3 and 6 months, respectively [27]. In the United Kingdom, 69% of mothers initiated BF at birth, but 21% and 36% discontinued the practice within the first 2 and 6 weeks, respectively [28]. In China, BF rates are higher than in Western countries, but vary between cities. The reported BF rates of cities from the northern to southern part of China from initiation to 4 months ranged from 54% and 37% in Beijing, 76.1% and 60.6% in Nanjing, and 94% and 56.6% in Guangzhou [29]. In Hong Kong, the initiation rate of BF was shown to be 74% in hospitals, but the EBF rate dropped to 24.1% at 6 weeks *postpartum* [30]. The public has been urged to increase breast feeding rates, with the argument that breast feeding offers optimal nutrition and natural protection to infants through antibodies passed on by the mother, and reduces the risk that the mother will develop breast cancer [31,32].

A systematic review conducted in 2012 revealed a trend of increase in atopic eczema between 1990 and 2010 in young children globally, including in some parts of Asia [9]. With the rise in atopy globally and with the controversy over the benefits of breast feeding and the early introduction of solid food in relation to atopy, there is a need to evaluate the evidence on the relationship between BF, the introduction of solid food, and atopic manifestations. Whether breast milk offers infants optimal immunity protection against atopy warrants investigation. The aim of this study is to explore the association between the type of feeding and the timing of the introduction of solid food, as well as maternal and prenatal confounding factors, family history, and environmental factors with the development of atopic illnesses in toddlers aged 4 months to 3 years.

## 2. Experimental Section

### 2.1. Study Design

This was a case-control study to examine the association between heredity, environment, and other confounding factors such as “type of feeding,” and atopic manifestations in toddlers aged 4 months to 3 years. The “cases” were toddlers with a medical diagnosis of atopy, while the “controls” were toddlers without atopy. The two groups of toddlers were compared in terms of their family history, environment, and types of feeding.

### 2.2. Subjects and Recruitment

The target population of this study was toddlers with or without atopy. The criteria for inclusion were toddlers between 4 months and 3 years of age who were born with no identifiable health issues, and who have or do not have atopy. Those who did not meet the inclusion criteria were excluded. Mothers with toddlers were recruited from the clinics of a private medical group in Hong Kong. This medical group has a total of three branches, which are located on Hong Kong Island, the south side of Hong Kong, and Lantau Island. The clinics provide pediatric health care services. Their clientele consists of mothers who bring their children in for scheduled check-ups or vaccinations, or to see a doctor if their child is unwell. The mothers were invited to join the study at the time of their visit.

All mothers with toddlers who fit the inclusion criteria were invited to participate in the study by completing a questionnaire on their child’s birth history, feeding mode, environmental exposure, family history, and atopic history. The mothers were given an explanation of the study and an information sheet on the study by a nurse working at the clinic. The mothers were clearly told that refusing to participate in the study would have no effect on the care that they receive from the clinic. Implied consent to participate in the study was given by those who agreed to complete the questionnaire developed for the study. A pediatrician in the clinic referred mothers of toddlers with known cases of atopy for participation in the study with a ticked box on the questionnaire to indicate the toddlers’ diagnosis of atopy. Known cases of atopy included diagnoses of eczema or allergic dermatitis, asthma, rhinitis, conjunctivitis (not related to infection), and allergies (dust mite, food, drug, *etc.*).

### 2.3. Development of the Questionnaire

A questionnaire was developed to solicit information on the birth history of the toddlers, the mother’s demographic data, the atopic history of the toddler and the toddler’s parents and siblings, and information on environmental factors. The questionnaire consisted of five sections:

Section 1: Birth history of the toddler, including gestational age, whether the toddler is singleton/twin, birth weight, and any health issue at birth (to confirm eligibility for inclusion);
Section 2: Mode of delivery (cesarean section or vaginal delivery), types of feeding (breast fed or formula fed, including duration; and introduction of solid food).
Section 3: Information on environmental factors, such as the history of maternal exposures, the hospitalization of the mother, maternal antibiotic use *in utero* or during breast feeding, the presence of siblings, carpets, pets, and so forth.
Section 4: Information on the parents’ atopic history, such as maternal self-reported or medically diagnosed allergic disease-atopic eczema, allergic rhinitis, asthma or food allergies, and so on.
Section 5: Information on the toddler’s atopic history, such as the occurrence of dermatological atopic manifestations, eczema, rhinitis, and other ailments, as well as history of hospitalization.

The questions in Sections 1 to 3 of the instrument were self-developed, based on a review of related literature. Section 4 consists of eight questions, which were modified from Lakwijk [33] The 16 questions in Section 5 were modified from the International Study of Asthma and Allergies in children (ISAAC) study. Three questions modified from Kilpeläinen [34] were added to this section.

The questionnaire went through a process of expert validation of the appropriateness and relevance of the questions. Five experts consisting of three pediatricians (one specializing in allergies) and two pediatric nurses were invited to validate the questionnaire. The content validity was calculated to be 0.91. A pilot test of the questionnaire was conducted among five mothers to determine whether they could understand the questions. The questionnaire was revised with larger bold font and wider spacing for easy reading. The mothers considered all of the questions to be comprehensible.

### 2.4. Data Processing and Analysis

The collected data were analyzed using SPSS Version 21.0 (IBM, Armonk, NY, USA). Descriptive statistics (frequency and percentages) were used to describe the demographics, characteristics of maternal and prenatal factors, environmental factors, and atopic manifestations of the toddlers. Toddlers were grouped according to those with a medical diagnosis of atopic disease and those without, and a statistical comparison was made of the toddlers’ demographic data and birth mode, the atopic history of their parents and siblings, environmental factors, and types of feeding. A *t*‑test was used to compare the parametric data, while all other nominal factors were compared using a chi-square test to determine statistical significance. Fisher’s exact test was used for groups with fewer than five subjects. All of the factors that had been identified as having statistical significance in relation to toddlers with atopic illness were included as independent variables in a logistic regression analysis performed to identify the contribution of factors of atopic illness in toddlers.

### 2.5. Ethical Consideration

Ethical approval was sought from the Human Subjects Ethics Sub-committee of The Hong Kong Polytechnic University. Mothers of toddlers were provided with a full explanation of the nature of the study, and it was confirmed that their participation was voluntary and that their non-participation would not affect the treatment received from physicians or nurses in the clinic. The completion of the questionnaire was considered a sign of implied consent. The participants were assured that their data would be kept confidential in accordance with the Privacy Ordinance. The anonymity of the individuals involved was ensured by the aggregation of statistical data. Access to the data was restricted to the researchers. Ethical approval was also sought and obtained from the governing body of the medical clinics, who were provided with copies of the research proposal and the questionnaire.

## 3. Results and Discussion

### 3.1. Results

During the data collection period from April to June 2014, 2,156 children visited the clinic, approximately 40% of them toddlers. Two hundred and six mothers of toddlers eligible for inclusion in the study completed the questionnaire. Due to the work flow of the clinic, the number of mothers who declined to participate or who were not eligible was not recorded.

#### 3.1.1. Characteristics, Birth History, and Mode of Feeding of the Toddlers

Table 1 shows information on the toddlers’ demographic data, birth history, and types of feeding. There were slightly more male (54.4%) than female toddlers (45.6%) in the study. The mean age of the toddlers was 20 months and their mean birth weight was 7.08 pounds.

**Table 1:** Demographic data, birth history, and mode of feeding of the children in the study.

Variable	*n* = 206 (%)	Medical Diagnosis of Atopic Disease (*n* = 71)	No Medical Diagnosis of Atopic Disease (*n* = 135)	*p*-Value (Chi-Square Test)
Gender		*n* (%)	*n* (%)	
Male	112 (54.4)	45 (63.4)	67 (49.6)	0.06
Female	94 (45.6)	26 (36.6)	68 (50.4)	
Age (months)	20.24 ± 11.795 SD	21.21 ± 11.009 SD	19.72 ± 12.200 SD	*t*-test 0.38
Birth weight (lbs.)	7.08 ± 1.468 SD	7.11 ± 1.570 SD	7.07 ± 1.418 SD	*t*-test 0.87
Mode of birth				0.74
Normal Vaginal	128 (62.1)	43 (60.6)	85 (63.0)	
Cesarean Section (Elective/Medical)	78 (37.9)	28 (39.4)	50 (37.0)	
Feeding Mode at Birth				
Exclusive Breast Feeding	155 (75.2)	58 (81.7)	97 (71.9)	0.12
Mixed Feeding	24 (11.7)	7 (9.9)	17 (12.6)	0.56
Exclusive Formula Feeding	27 (13.1)	6 (8.5)	21 (15.6)	0.15
Exclusive Breast Feeding for 3 months	131 (63.6)	52 (73.2)	79 (58.5)	0.04 *
Exclusive Breast Feeding for 6 months	55 (26.7)	20 (28.1)	35 (25.9)	0.73
Age at commencement of mixed Feeding (days old) (*n =* 92)	113.59 ± 137.225 SD	161.59 ± 191.520 SD	87.98 ± 88.628 SD	*t*-test 0.05 *
Age when solid food was first introduced (weeks)	25.22 ± 9.287 SD	24.04 ± 6.675 SD	25.90 ± 10.481 SD	*t*-test 0.15

Notes: * = *p* ≤ 0.05

Approximately two-thirds (62.1%) of the toddlers had been born by vaginal delivery and one-third (37.9%) by cesarean section (CS). Fifteen percent of the births by CS were medically necessary. More than one-third of the toddlers (71 out of 206; 34.5%) in this study were reported to have received a medical diagnosis of atopic disease. Table 1 gives a comparison of the toddlers who had been diagnosed with and without atopy.

Mode of birth was not found to be associated with atopy in these toddlers. While the majority of toddlers (75.2%) were breast fed exclusively at birth, the percentages decreased to 63.6% and 26.7% at 3 and 6 months, respectively. Only a small percentage of toddlers were formula fed (13.1%), and fed with both breast and formula milk (11.7%) since birth.

More of the atopic toddlers were exclusively breast fed for 3 months (73.2%) than those who were non-atopic (58.5%), and the difference between the groups was statistically significant (*p* < 0.04). The toddlers in this study started mixed (breast milk and formula) feeding at an average of 113 days old (16.1 weeks). The toddlers with atopic illness were shown to have started mixed feeding much later, at 161 days old (23 weeks), compared to the non-atopic toddlers, who commenced at approximately 88 days old (12.6 weeks). There was statistical significance (*p* < 0.05) with the time of the commencement of mixed feeding. The mean age at which the toddlers had their first solid food was approximately 25 weeks (around 5.8 months). No association was found between atopy and the age at which the toddlers were introduced to solid food.

#### 3.1.2. Family History of Atopic Disease

Among those with atopic disease, the most common types of atopic disease were eczema/dermatitis (67.6%) and itchy rash (29.6%) (Table 2). Table 2 shows the family history of atopic disease of the toddlers in the study. A family history of atopic disease was common among the 206 toddlers in the study, with 68 (33.0%) of them having one parent who had been diagnosed atopic disease, while 22 (10.7%) had both parents who had received such a diagnosis; among those with siblings (*n =* 115), 50 (43.5%) had a sibling who had been diagnosed with atopic disease. Among the mothers, the most common types of atopic disease were eczema (37.5%), asthma (34.4%), and hay fever (34.4%). Among the fathers, the most common types were also eczema (35.4%), asthma (31.3%), and hay fever (29.2%). Among the siblings, the most common types of atopic disease were eczema (48%), itchy rash (22%), and rhinitis (20%). In Hong Kong, eczema was the most prevalent atopic disease among all family members, with approximately 20% of toddlers having both parents who had suffered from eczema. The results of the chi-square test showed statistically significant differences between toddlers with and without atopic disease. The differences are: both parents with atopic history (*p <* 0.01), maternal history of eczema (*p <* 0.01), maternal history of itchy rash (*p <* 0.02), father diagnosed with atopy (*p <* 0.01), paternal history of asthma (*p <* 0.03), paternal history of food allergies (*p <* 0.01), and siblings with a history of all of types of atopic disease except for food allergies.

**Table 2:** Family history of atopic disease of the children who participated in the study.

Family Member	*N =* 206 %	Dx of Atopic Disease (*n =* 71) n (%)	No Dx of Atopic Disease (*n =* 135) *n* (%)	*p*-Value (Chi-Square Test or Fisher’s Exact Test *)
Children diagnosed with atopy		71 (100)		
*Types of diagnosis*				
Eczema/Allergic dermatitis		48 (67.6)		
Itchy rash		21 (29.6)		
Rhinitis		13 (18.3)		
Asthma		9 (12.7)		
Conjunctivitis (noninfection)		12 (16.9)		
Food allergies		11 (15.5)		
Dust mite allergy		9 (12.7)		
Positive allergy test		7 (9.9)		
Pet allergy		4 (5.6)		
Drug allergies		2 (2.8)		
Not specified		2 (2.8)		
Parental history of atopic disease				
None	116 (56.3)	35 (49.3)	81 (60.0)	0.14
One parent	68 (33.0)	23 (32.4)	45 (33.3)	0.61
Both parents	22 (10.7)	13 (18.3)	9 (6.7)	0.01 **
Mother diagnosed with atopy	64 (31.1)	25 (35.2)	39 (28.9)	0.35
*Types of diagnosis*				
Eczema/Allergic dermatitis	24 (37.5)	15 (60.0)	9 (23.1)	<0.01 **
Itchy rash	10 (15.6)	7 (28.0)	3 (7.7)	0.02 *
Rhinitis	13 (20.3)	7 (28.0)	6 (15.4)	0.13
Asthma	22 (34.4)	7 (28.0)	15 (38.5)	0.78
Hay fever	22 (34.4)	9 (36.0)	13 (33.3)	0.50
Food allergies	9 (14.1)	3 (12.0)	6 (15.4)	0.94
Pet allergies	17 (26.6)	7 (28.0)	10 (25.6)	0.54
Dust mite allergy	21 (32.8)	8 (32.0)	13 (33.3)	0.71
Others	6 (9.4)	4 (16.0)	2 (5.1)	0.09
Drug allergies	5			
Father diagnosed with atopy	48 (23.3)	24 (33.8)	24 (17.8)	0.01 **
*Types of diagnosis*				
Eczema/Allergic dermatitis	17 (35.4)	5 (20.8)	12 (50.0)	0.65
Itchy rash	3 (6.3)	2 (8.3)	1 (4.2)	0.24
Rhinitis	10 (20.8)	5 (20.8)	5 (20.8)	0.29
Asthma	15 (31.3)	9 (37.5)	6 (25.0)	0.03 *
Hay fever	14 (29.2)	8 (33.3)	6 (25.0)	0.06
Food allergies	4 (8.3)	4 (16.7)	0 (0)	<0.01 **
Pet allergies	14 (29.2)	6 (25.0)	8 (33.3)	0.49
Dust mite allergy	12 (25.0)	7 (29.2)	5 (20.8)	0.07
Others	5 (10.4)	2 (8.3)	3 (12.5)	0.79
Drug allergies	3			
Not specified	2			
Siblings diagnosed with atopy (only among those with siblings *n =* 115)	50 (43.5)	24 (33.8)	26 (19.3)	0.02 *
*Types of diagnosis*				
Eczema/Allergic dermatitis	24 (48.0)	11 (45.8)	13 (50.0)	0.03 *
Itchy rash	11 (22.0)	6 (25.0)	5 (19.2)	0.027 *
Rhinitis	10 (20.0)	7 (29.2)	3 (11.5)	<0.01 **
Asthma	8 (16.0)	6 (25.0)	2 (7.7)	<0.01 **
Hay fever	6 (12.0)	5 (20.8)	1 (3.8)	<0.01 **
Food allergies	8 (16.0)	4 (16.7)	4 (15.4)	0.06
Pet allergies	5 (10.0)	4 (16.7)	1 (3.8)	<0.01 **
Dust mite allergy	9 (18.0)	5 (20.8)	4 (15.4)	0.03 *
Others	2 (4.0)	2 (8.3)	0 (0)	0.01 **
Drug allergy	1			
Not specified	1			

Notes: * = *p* ≤ 0.05, ** = *p* ≤ 0.01; * Fisher’s exact test used when ˂ 5 subjects within a group.

#### 3.1.3. Environmental Exposures of Toddlers

Table 3 shows the environmental exposures of the toddlers. There was no statistical significance between the toddlers with atopic disease and those without in terms of their environmental exposure to siblings, pets, carpeting, plants, cigarette smoke, pre-school or play groups, and maternal hospitalization during pregnancy or maternal smoking (or passive smoking). The only statistically significant difference between the two groups of toddlers was in antibiotic use *in utero* or during breast feeding (*p <* 0.02), with slightly more of those toddlers who were exposed being atopic (35.2%) than non-atopic (20.7%)s.

**Table 3:** Environmental exposure of those toddlers in the study with and without the diagnosis of atopy.

Environmental Factors	*N =* 206%	Dx Atopic Disease (*n =* 71)	No Dx of Atopic Disease (*n =* 135)	*p*-Value (Chi-Square Test)
**Factors Associated with Toddlers**		***n* (%)**	***n* (%)**	0.48
Siblings	115 (55.6)	37 (52.1)	78 (57.8)	0.44
Carpets	57 (27.7)	24 (33.8)	33 (24.4)	0.15
Pets	35 (17.0)	14 (19.7)	21 (15.6)	0.45
Plants	78 (37.9)	28 (39.3)	50 (37.0)	0.74
Smoke in household	33 (16.0)	11 (15.5)	22 (16.3)	0.88
Preschool/playgroup	114 (55.3)	40 (56.3)	74 (54.8)	0.83
**Maternal Exposures**				0.08
Hospitalization during pregnancy	33 (16.0)	11 (15.5)	22 (16.3)	0.88
Smoking in or near pregnancy	28 (13.6)	11 (15.5)	17 (12.6)	0.56
Antibiotic use (*In utero* or during breastfeeding)	53 (25.7)	25 (35.2)	28 (20.7)	0.02 *

Note: * = *p* ≤ 0.05.

#### 3.1.4. Factors Contributing to Atopic Disease in Toddlers

All of the factors found in the previous analysis to be of statistical significance with regard to the differences between toddlers with and without atopy were included in the logistic regression analysis to identify the predictive factors of atopy. These factors were: exclusively breast fed for 3 months, commencement of mixed feeding (at ≥ 92 days of age), mother, father, and siblings diagnosed with atopic disease, and antibiotic use *in utero* or during pregnancy. Table 4 shows the results of the binary logistic regression analysis, which indicate that atopy is associated with the male gender (OR 2.072, CI 1.089–3.941), maternal antibiotic use *in utero* or while breast feeding (OR 2.276, CI 1.151–4.504), the later commencement of mixed feeding (OR 2.497, CI 1.025–6.082), exclusive breast feeding for 3 months (OR 1.972, CI 1.009–3.857), and a maternal diagnosis of eczema (OR 4.510, CI 1.764–11.530). These findings (factors contributing to atopic disease) are discussed below.

**Table 4:** Factors contributing to atopic disease in toddlers, by binary logistic regression.

Variable (Reference Group)	Odds Ratio	95% CI
Gender *(female)*	2.072	1.089–3.941
Maternal antibiotic use *in utero* or while breastfeeding *(Non-exposure)*	2.276	1.151–4.504
Time of commencement of mixed feeding *(commenced earlier at ≤91 days)*	2.497	1.025–6.082
Exclusively breastfed for 3 months *(breast fed for less than 3 months)*	1.972	1.009–3.857
Mother diagnosed with eczema *(without diagnosis)*	4.510	1.764–11.530

### 3.2. Discussion of the Major Findings of the Study

#### 3.2.1. Mode of Delivery and Types of Feeding

A review of the population showed an increase in the percentage of women who reported that they had undergone a CS, which may be due to a preference for CS among Hong Kong (HK) women. A study of HK women showed that 16.7% of women who had previously had a CS delivery would prefer to undergo a CS, while the rate for first-time mothers was 16.8% [35]. The reasons given by mothers for preferring a CS were better control of time, fear of vaginal birth, worries about pain, the perception that CS as a safer method of birth for the baby, less vaginal trauma, and if the baby had been conceived by *in vitro* fertilization.

As no association was found between mode of delivery and atopy, the result contradicts that of a birth cohort study in United States that found that atopic illness is associated with cesarean section delivery [36]. However, it must be noted that the parents of the children included in the US study had a history of atopic illness, which may account for the association.

The EBF rates of the women in this study were higher than those reported by the HK government, which found that 19%, 18%, and 15% of infants were exclusively breastfeeding at one, two, and four to six months, respectively [37]. The improvement in BF rates may be due to strenuous efforts on the part of the government in the past few years to promote breast feeding [37]. The HK government developed and implemented advisory guidelines in 2008 on the provision of baby care rooms in public facilities and improved baby care facilities in government offices [37]. In 2013, improvements in breast feeding rates were reported [37]. In the past few years, other organizations such as La Leche League and UNICEF HK have also participated in the promotion of breast feeding [38]. A comparison of EBF rates at 6 months with those of other countries such as the United States (13%) [27] and United Kingdom (1%) [39] revealed higher rates in Hong Kong. The age at which toddlers in Hong Kong were first exposed to solid food was in line with the recommendations made by the Department of Health [31] and the World Health Organization [40] that solid foods be introduced at 6 months of age.

#### 3.2.2. Eczema/Allergic Dermatitis as the Dominant Atopic Disease

In this study eczema was identified as the most common type of atopy among toddlers. This differs from a previous study conducted in HK, which found rhinitis to be a more prevalent atopic disease than eczema; however that study was conducted among older children, aged from 13 to 14 years [41]. Our finding is consistent with that of a Swedish study on a 1979 birth cohort that was followed to 1991 [42]. Other studies have also identified eczema (atopic dermatitis) as the most common atopic skin disorder among children [43]. However, another study argued that no one type of atopy is prevalent over others, but that the situation varies among regions [44].

The prevalence of eczema in Hong Kong in the past decades has also fluctuated. A comparison of studies reveals a noticeable per year increase of 0.12% in the prevalence of atopy in HK from 1995 to 2001, and a reported rate of current eczema of 4.6% in young children in 2001 [45]. In 2006, reports of current eczema in young children were 5.8% [46], rising to 15% in 2011 [1].

Studies on atopic dermatitis (eczema) involving twins and those with a family history of the disease have shown that the predisposition to atopic dermatitis is highly hereditary [47]. Family history also contributes to the development of other types of atopies, such as asthma [48]. Multiple factors have been shown to contribute to the occurrence of eczema in childhood, including antibiotic use in early life [49] or *in utero* by the mother [50,51], and a paternal and maternal history of eczema [52]. Those with a family history of atopic dermatitis have been shown to have a high chance of passing on the condition to their offspring [47].

#### 3.2.3. Atopic Disorders Dominant in the Male Gender

Male toddlers (63.4%) were found to be about twice as likely (odds ratio of 2.072; *p =* 0.03) than female toddlers to develop atopic diseases up to the age of three (Table 1 and Table 4). The male gender has previously been identified as a genetic and lifestyle risk factor in the development of atopy [53,54]. Ferraz *et al.* [53] and Moore *et al.* [54] investigated the risk of the male gender in relation to atopic dermatitis and sensitization to a number of allergens, respectively. Similar studies also found males to be more susceptible to asthma in childhood than females [55,56], possibly because male children have a smaller airway in portion to their lung size than with females, making them more sensitive to infections and airborne triggers [57], in turn leading to greater sensitivity to aeroallergens [58]. Interestingly, another study conducted in Germany [59] reported that a non-atopic type of eczema occurred more frequently among preschool girls than boys. In our study, the male gender was identified as a risk factor for atopy among toddlers.

#### 3.2.4. Maternal Antibiotic Use *in Utero* or While Breastfeeding as a Key Environmental Factor Associated with Atopy

Maternal antibiotic use *in utero* or while breast feeding has been identified as a significant environmental factor in toddler atopy, with an odds ratio of 2.276 (Table 3 and Table 4). The findings are consistent with those of other studies on antibiotic use *in utero*, which found antibiotic use to be a risk factor for atopic disease in children, in relation to asthma [50,51] and eczema [51]. As early as 1999, the hypothesis was put forward that exposure to infections in early life and gut colonization of bacteria protects against atopy [60]. The association between atopic disease and exposure to antibiotics was accounted for by either interference with the composition of commensal microflora or the alteration of the course of bacterial infections [61]. However, exposure to antibiotics during pregnancy or breast feeding may be frequent due to treatment for infections such as mastitis [61]. Studies have found that exposure to antibiotics during these periods puts children at a risk of developing atopic diseases such as asthma [50,51], eczema [51], and recurrent wheezing [61]. However, antibiotic use may be unavoidable because of the need to treat infections. There is a need to weigh the benefits of using antibiotics in relation to the risks, and to prevent antibiotics from being over- prescribed.

#### 3.2.5. Late Introduction to Mixed Feeding and Prolonged Exclusive Breast Feeding as Risk Factors for Atopic Disorders

The later commencement of mixed feeding at ≥ 92 days 2.497 (*p <* 0.05) and EBF for 3 months 1.972 (*p <* 0.05) was found to be associated with atopic disease in toddlers (Table 4). There is no recent study on the association between mixed feeding (BF with formula feeding) and atopy. However, a study conducted in 1980 that measured serum IgE levels in toddlers and compared them with the time at which the toddlers were weaned with cow’s milk found the levels to be significantly higher in infants who started weaning later than 6 months than in infants who were weaned before 6 months of age [62]. It was also found that the earlier at which a toddler began to be fed with cow's milk, the lower the level of IgE; and the later the cessation of breast feeding, the higher the level of IgE [62]. These findings are consistent with our finding that a late introduction to mixed feeding is a risk factor for atopy. An earlier introduction to an alternate form of feeding may work to increase food diversity or microbial exposure, which may protect against atopy [13].

The finding of this study on exclusive breast feeding is consistent with that of previous studies, which found that exclusive breast feeding for long durations increases the risk of developing atopic illnesses such as asthma [21] and eczema or atopic dermatitis [18,19,20]. These studies examined durations of exclusive breast feeding, ranging from 4 weeks to 6 months, in countries such as Finland, Denmark, New Zealand, and Germany. Many studies, including a meta-analysis conducted in 2009, have found no strong evidence to support the notion that EBF has a protective effect against atopies in children [21,22,23]. Surprisingly, our results showed that there was no statistical significance between EBF and atopy at 6 months, but that the situation was different at 3 months (*p <* 0.05). A possible explanation for this is that an infant’s immune system is immature at birth and partially dependent on its mother’s antibodies for the protection acquired through breast feeding [63]. The infant is sensitized by the presence of antibodies in the breast milk of its mother and manifests as atopic. As an infant matures and starts to produce its own antibodies, the infant recovers from atopic sensitization [64]. An example of this would be food allergies in children, where foods to which children displayed allergic reactions earlier in their first year of life, can be successfully re-introduced few months later from the onset of the allergy [65].

#### 3.2.6. Maternal Atopic History Identified as a Strong Contributing Factor

The findings of this current study are consistent with studies that found a maternal history of eczema to be a risk factor for atopic dermatitis in children, and also to be a more predictive factor of eczema than a paternal history of the condition [54,66]. A maternal history of eczema was found to be the strongest risk factor, with an odds ratio of 4.510 (*p* < 0.01), among the five significant factors shown in Table 4. It is logical to postulate that a mother with an atopic history would carry specific antibodies against antigens that trigger the atopy, and that such antibodies would be present in her breast milk. It is also known that atopic dermatitis is highly hereditary, in that having family history or twins with the condition is strongly related to the occurrence of atopic dermatitis in infants [47].

#### 3.2.7. Is the Quality of Breast Milk a Cause of Atopic Diseases in Toddlers?

Most types of exposure, whether to something in the environment or to types of feeding, trigger a tolerogenic effect on the immune system mediating towards the non-disease state; however some types of exposure can cause a dysregulation of this cycle and a mediation towards an allergic response, causing atopy [17]. Some published theories relating to immunity could explain this phenomenon. The first is the “hygiene hypothesis”, in which it is claimed that limiting early-life infections hinders the natural development of the immune system and causes a predisposition to allergic diseases [67]. The other is the “microflora hypothesis”, which states that a Western lifestyle leads to the creation of an overly “hygienic society”, which limits general microbial exposure and alters the colonization of the infant gut, which in turn disrupts the development of the immune system and ultimately leads to allergic diseases [68].

There is evidence that breast milk with a certain content may be the underlying cause of atopy in children. Breast milk containing antigens [69], cytokines [70], and fatty acid n-3 transmitted from the mother through breast feeding has been found to cause an allergic sensitization [70] to foods [69], or to lead to the eventual development of atopy and aerosensitization [71] in infants. However, the mothers involved in these studies had a history of atopy, suggesting that this history, and the content of their breast milk, could have been the cause of the atopy in the infants. The content of breast milk warrants further investigation. Immunological studies should be conducted to investigate allergic factors such as antigens, antibodies, and even types of microflora in the breast milk produced by atopic mothers, to examine the relationship between these factors and the development of atopy in children.

### 3.3. Limitations

This study was not without limitations. First of all, the subjects in this study were recruited from a private medical clinic and thus can be assumed to be more affluent and from higher socio-economic class than the general population. Second, this was a cross-sectional study to examine the association of factors, and thus it was not possible to examine changes in the atopic status of the infants over time.

## 4. Conclusions

Atopy in children can be multifactorial. The associating risk factors identified were being boys, later commencement of mixed feeding, exclusive breast feeding for 3 months, mother diagnosed of eczema, and maternal antibiotic use in-utero or while breast feeding. The maternal factors were identified as contributing factors to atopic disease in toddlers in that maternal antibiotics use of mothers and diagnosed of eczema of mothers. The results from this study warrants further investigation into possibly the content of breast milk, including the comparison of the breast milk content of those mothers who are atopic with those who are not.
